# Determining the heat of desorption for cassava products based on data measured by an automated gravimetric moisture sorption system

**DOI:** 10.1002/jsfa.12153

**Published:** 2022-09-01

**Authors:** Hamed J Sarnavi, Marcelo Precoppe, Pablo García‐Triñanes, Arnaud Chapuis, Thierry Tran, Michael SA Bradley, Joachim Müller

**Affiliations:** ^1^ Natural Resources Institute, Faculty of Engineering and Science University of Greenwich Chatham UK; ^2^ Materials and Chemical Engineering Group, School of Engineering University of Greenwich Chatham UK; ^3^ CIRAD, UMR Qualisud Saint‐Louis Sénégal; ^4^ Qualisud, Université de Montpellier, CIRAD, Montpellier SupAgro, Université d'Avignon, Université de La Réunion Montpellier France; ^5^ CGIAR Research Program on Roots Tubers and Bananas (RTB) The Alliance of Bioversity International and the International Center for Tropical Agriculture (CIAT) Cali Colombia; ^6^ Wolfson Centre for Bulk Solids Handling Technology, Faculty of Engineering & Science University of Greenwich Chatham UK; ^7^ Tropics and Subtropics Group, Institute of Agricultural Engineering University of Hohenheim Stuttgart Germany

**Keywords:** flour, starch, sorption isotherm, thermodynamic properties, drying efficiency

## Abstract

**BACKGROUND:**

The isosteric heat of desorption is vital in evaluating the energy performance of food dryers. The isosteric heat of desorption was investigated for different cassava (*Manihot esculenta* Crantz) products prepared as flour or starch, with and without fermentation. An automated moisture sorption gravimetric analyser was used to measure the desorption isotherms over 10–90% relative humidity of the drying air at temperatures ranging from 25 to 65 °C.

**RESULTS:**

Analysis of variance showed an imperceptible contribution of the preparation method in the measured desorption data. This finding also agreed with microscopical images, which revealed the lack of compelling structural differences among different products. A set of empirical sorption equations suggested by the ASAE standard was examined over the measured desorption isotherms. The standard error of estimation was found to be in the acceptable range of 2.36–3.71%. Furthermore, the fulfilment of the enthalpy‐entropy compensation theory was considered as an additional criterion in the thermodynamic results of different sorption equations, besides their fitting adequacy. The modified Chung–Pfost equation has proved to be the most suitable equation for cassava products, as it is capable of reflecting the temperature dependency of the isosteric heat of desorption. The net isosteric heat of desorption obtained was in the range of 540–1110 kJ kg^−1^ for 0.10 kg kg^−1^ dry‐basis moisture content and 52–108 kJ kg^−1^ for 0.25 kg kg^−1^ dry‐basis moisture content.

**CONCLUSION:**

These findings are technologically relevant for optimising common drying technologies such as flash and flatbed dryers. © 2022 The Authors. *Journal of The Science of Food and Agriculture* published by John Wiley & Sons Ltd on behalf of Society of Chemical Industry.

**ACRONYMS AND ABBREVIATURES**
ANOVA: Analysis of variance
EMC: Equilibrium moisture content
ERH: Equilibrium relative humidity
SEM: Scanning electron microscopy
GAB: Guggenheim‐Anderson‐de Boer
MRPE: Mean Realtive Percentage Error
SEE: Standard Error of Estimation
kg kg^−1^ db: Kilograms of water per kilogram dry matter
M
: Moisture content (kg kg^−1^ db)
$\hat{\mathrm{ERH}}$
: Predicted ERH (decimal values)
T
: Temperature (°C)
$T^{\mathrm{abs}}$
: Absolute temperature (K)
$T_{\beta }$
: Isokinetic temperature (K)
$T_{\mathrm{hm}}$
: Harmonic mean temperature (K)
$p_{i}$
: Parameters of the sorption equations in Table 1
$L_{v}$
: Heat of vaporisation for pure water (J mol^−1^ water)
$Q_{\mathrm{st}}$
: Isosteric heat of desorption (J mol^−1^ water)
$q_{\mathrm{st}}$
: Net isosteric desorption heat (J mol^−1^ water)
${\dot{Q}}_{\text{in}}$
: Input energy rate of the dryer (kJ h^−1^)
${\dot{m}}_{w}$
: Water evaporation rate in the dryer (kg h^−1^)
$W_{w}$
: Weight of a water molecule (kg mol^−1^ water)
$X_{\mathrm{ini}}$
: Initial moisture content of the batch in the dryer (kg kg^−1^ db)
∆X
: Reduction in moisture content in the specific time interval of ∆t
$W_{\mathrm{ini}}$
: Initial weight of the batch in the dryer (kg)
η
: Energy efficiency of the dryer
R
: Universal gas constant
∆H
: Differential enthalpy (J mol^−1^ water)
∆S
: Differential entropy (J mol^−1^ water K^−1^)
$∆G_{\beta }$
: Differential Gibbs free energy at isokinetic temperature (J mol^−1^ water)

## INTRODUCTION

Cassava (*Manihot esculenta* Crantz), a perennial tuberous plant with high‐starch‐content roots, has been gaining dramatic importance in the global food market in recent decades.^1^ Despite all its advantages, cassava is subject to severe challenges impacting its production, consumption and economics. Among them, rapid postharvest physiological deterioration of the root has been mentioned as the major constraint affecting the economic value of this crop.^2^ Thus many different ways are used to process cassava, most of which result in a dried product with a moisture content below 0.14 kg kg^−1^ db.^3^ The cassava root can be consumed raw or processed, fermented or unfermented, in the form of starch or flour, either as an ingredient or as an additive, among other products. In general, the roots are peeled, grated, pressed, dried and milled. Additionally, it is common to apply other techniques such as fermentation, detoxifying the cyanogenic glycosides, and enhancing the aromatic features of cassava. In West and Central African regions, grated cassava roots are fermented in sacks for 3–5 days, encouraging lactic acid fermentation and a consequent reduction in pH value to less than 4.0.^4^

Fixed‐bed and pneumatic dryers are the primary dryers used by small‐sized African enterprises to produce cassava flour.^5^ They are often mis‐dimensioned and use up to four times more energy than necessary, with financial and environmental repercussions for cassava value chains.^6^ In designing equipment for the drying process, the heat of sorption represents the energy needed to remove a specified amount of water from a product with a particular water content. According to the energy input, the energy efficiency can be calculated using the heat of sorption.^7^ It is therefore essential to determine the differential heat of sorption, and its variation as food is dehydrated to low moisture levels.^8^ Implementing the Clausius–Clapeyron analysis on the experimental data of the isosteric equilibrium pressures at various temperatures determines the differential enthalpy, also known as the isosteric heat of sorption.^9^ The experimental sorption isotherms can be represented by different sorption equations on either a theoretical or empirical basis. Some of those more commonly used are discussed in other reports.^8^, ^10^, ^11^ The criteria for selecting the most appropriate sorption equation are usually the degree of fit to the experimental data and the model's simplicity.^8^, ^10^, ^11^, ^12^, ^13^ Empirical models are usually used when theoretical equations, such as Guggenheim–Anderson–de Boer (GAB),^8^, ^10^, ^11^ could not fit data over the entire range of equilibrium moisture content (EMC). This can be attributed to the fact that water is associated with the food matrix by different mechanisms at different levels of water content. The parameters of the GAB equations must have physical meanings as they are defined based on physical adsorption phenomena.^14^ In contrast, the parameters of the empirical equations can take any values without limitations as they have no physical implications. Thus they could give a better fit quality over a broader range of experimental data.

Thermodynamic characterisations are used to understand the properties of the embedded water inside the food material by relating its partial pressure and concentration and calculating energy requirements associated with heat and mass transfer occurring in drying systems.^15^ Differential enthalpy decreases slightly with temperature. Higher temperatures will cause a corresponding decrease in differential entropy, reducing the number of adsorbed molecules.^11^ The Gibbs–Helmholtz equation can also be used to calculate the change in molar differential entropy of desorption. Changes in both enthalpy and entropy usually accompany the free energy changes resulting from water sorption.

Moreover, the isokinetic theory or enthalpy–entropy compensation theory is widely used to evaluate physical phenomena such as desorption in some starchy materials.^16^ These characteristics also provide an insight into the microstructure of the water and food matrix^11^ and determine the minimum energy required to remove a specific amount of water from the food.^17^ Hence it usually comes with further relevant information on the product's microstructural properties, such as those obtained by scanning electron microscopy (SEM) photographs, to describe and characterise the sorption process based on the granular morphologies of cassava.^18^, ^19^

Three basic techniques, namely manometric, gravimetric and hygrometric, are used to determine the sorption isotherms of agricultural materials.^8^, ^13^ The gravimetric technique is the most common one,^13^ and has already been used for cassava products.^17^, ^20^, ^21^, ^22^, ^23^, ^24^ However, none of the previous works has employed dynamic vapour sorption systems for cassava products. The static gravimetric technique encounters some problems at high humidity ranges due to excessive equilibration times and the inability to supply and maintain high relative humidity values.^13^ Additionally, when incubating cassava samples in the presence of salt solutions for a period of weeks using the static gravimetric technique, they can be prone to deterioration, which often begins within only 24–72 h following harvest.^4^ Therefore, the rapidity of the measurements with dynamic vapour sorption systems can avoid errors linked to cassava sample deterioration, which can occur during incubation in the presence of salt solutions over weeks, for example. Automated climate chambers have been employed to measure the equilibrium moisture content of the material at any desired relative humidity and selected temperatures in a short time.^25^, ^26^ This automated system can provide quicker and more accurate measurements over a more extensive temperature range, thus yielding data that are suitable for modelling an entire cassava drying process. Furthermore, the temperature dependency of the sorption heat was not considered in previous works. However, to obtain a correct energy balance and realistic simulations of the cassava drying process, reliable temperature‐dependent values for the heat of sorption are required.

Therefore, this research used an automated gravimetric moisture sorption system, as described previously,^26^ to measure desorption data for pulverised cassava grits. Four different cassava products, commonly prepared in the West and Central African regions, were studied. The main objective of this study was to determine the net isosteric heat of sorption as a function of the product water content and the drying air temperature. The fit quality was examined for a set of empirical sorption equations suggested by ASAE D245.7.^27^ Furthermore, the ability to justify the compensation theory was considered an additional criterion for choosing the best sorption equations for Clausius–Clapeyron analysis. Finally, it was shown how the findings could contribute to evaluating the energy efficiency of a convection‐based dryer designed for cassava products.

## MATERIAL AND METHODS

### Material preparation

Cassava roots were purchased from the local market. Flour samples were prepared by blending a mixture of 250 mL distilled water and 250 g sliced cassava roots for 5 min in a kitchen blender. For preparing the fermented flour, the blended mixture was placed in an incubator for 3 days at 35 °C. It was kept in a lidded pot. The mixture was mechanically dewatered with a tincture press at 20 kPa of pressure. The sample had 0.45 kg kg^−1^ wet‐based moisture content after pressing. Starch samples were also prepared by blending a mixture of 300 mL distilled water and 300 g sliced cassava roots for 5 min in the same kitchen blender. This was repeated twice. The mixture was washed by adding step‐by‐step distilled water, first through a sieve with 168 μm pore size and then a sieve with 63 μm pore size. For fermented starch, the obtained filtrate was put into an incubator at 30 °C for 3 days. In contrast, unfermented starch was kept in the fridge between 4 and 6 °C for 3 days. After discarding the supernatants, they were centrifuged at 13 500 rpm for 20 min to discard further supernatants. The samples were kept individually in airtight thermostatic containers to homogenise the temperature and water content distribution through the sample before the moisture desorption tests. According to a previous report,^28^ the proximate composition of cassava flour was expected to be 1.5 g for protein, 0.2 g for fat, 2.5 g for fibre, 1.8 g for ash and 83.8 g for carbohydrate per 100 g cassava.

### Moisture desorption isotherm tests

**Figure 1:**
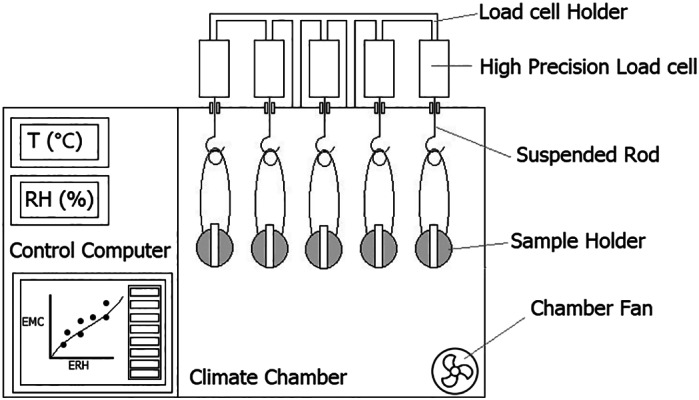
The automated system built for dynamic vapour sorption recording at the Institute of Agricultural Engineering, University of Hohenheim, Stuttgart, Germany.^26^ and used for measuring the desorption isotherms of the cassava products. Drawing is not to scale.

The desorption isotherms of cassava flour and starch, fermented and unfermented, were separately determined using an automated system designed at the Institute of Agricultural Engineering, University of Hohenheim (Stuttgart, Germany)^26^ (Fig. 1). The prepared samples of cassava products stored under room conditions (25 °C and 60% relative humidity) were loaded into the sample holder embedded in a climatic test chamber (C + 10/600, CTS GmbH, Hechingen, Germany) equipped with a temperature‐controlled humidification system. The sample holder consists of five high‐precision load cells (WZA1203‐N, Sartorius AG, Goettingen, Germany) with an accuracy of 0.001 g, mounted on top of the climate chamber to record the change in mass caused by desorption of water vapour. Equilibrium was considered to have been reached when the change in mass was less than 0.001 mg min^−1^. The system is computer‐controlled, allowing stepwise decrease of relative humidity from 85% to 10% in decrements of 15% at a set temperature and continuous measurement of temperature, humidity and mass during the sorption process. The desorption isotherms were determined at five temperature levels: 25, 35, 45, 55 and 65 °C. Regarding good reproducibility, sorption experiments were repeated twice for each sample.

### SEM analysis

The micromorphology of cassava products was photographed using a scanning electron microscope (TM3030 Plus benchtop electron microscope, Hitachi, Tokyo, Japan. After ethanol soaking, the samples were glued to the silver tape connected to the brass plate, followed by vacuum coating with gold. SEM images were taken after undergoing a desorption process. The observations were conducted under an accelerating potential of 1200 V and photographed at several magnifications. The final images were obtained from the SEM image acquisition software.

### Data analysis

Statistics were done using R.^29^ Data obtained from the sorption tests were submitted to analysis of variance, at 5% significance, using the R functions of ‘aov’. The ‘nls’ function was also used when the multi‐temperature desorption data set was subjected to nonlinear regression analysis. The Gauss–Newton algorithm with convergence criterion of $10^{-6}$ was implemented, minimising the sum of squares of the desorption equation's deviations in a series of iterative steps.

### Desorption isotherm modelling

The sorption equations recommended by ASAE standard^27^ (Table 1) were used to describe the relationship between the set equilibrium relative humidity (ERH) values and their corresponding measured equilibrium moisture content (EMC) values at different levels of air temperatures. Their constants are obtained by conducting a nonlinear regression analysis on experimental values. The coefficient of determination (*R* ^2^), calculated as Eqn (1), provides a classic measure of how well the sorption equations mimic the input ERH values. It can be interpreted as the proportion of the variance in the predicted ERH values attributable to the variance in the actual ERH values. The mean relative percentage error (MRPE), calculated as Eqn (2), and the standard error of estimation (SEE), calculated as Eqn (3), were also considered as objective criteria of fitness.

(1) $$R^{2}=1-\frac{\sum_{i}^{N}{\left({\mathrm{ERH}}_{i}-{\hat{\mathrm{ERH}}}_{i}\right)}^{2}}{\sum_{i}^{N}{\left({\mathrm{ERH}}_{i}-\frac{\sum_{i}^{N}{\mathrm{ERH}}_{i}}{N}\right)}^{2}}$$

(2) $$\text{MRPE}=\frac{100}{N}\sum_{1}^{N}\left|\frac{{\mathrm{ERH}}_{i}-{\hat{\mathrm{ERH}}}_{i}}{{\mathrm{ERH}}_{i}}\right|$$

(3) $$\mathrm{SEE}=\sqrt{\frac{\sum_{i}^{N}{\left({\mathrm{ERH}}_{i}-{\hat{\mathrm{ERH}}}_{i}\right)}^{2}}{\mathrm{df}}}$$

where N is the size of the data subset, $\hat{\mathrm{ERH}}$ is the predicted value for ERH, and df is the degree of freedom equal to *N* subtracted by the number of estimable parameters. Having studied all these indices, the goodness of fit of a sorption model to experimental data could show only a mathematical quality and not the nature of the sorption process.^11^

**Table 1:** Empirical sorption equations suggested by ASAE standard (ASAE D245.7, 2021)

Equation name	Mathematical expression
Modified Chung–Pfost	$\mathrm{ERH}=\exp\left[\frac{-p_{1}\;}{T+p_{3}}\exp\left[-p_{2}\mathrm{EMC}\right]\right]$
Modified Halsey	$\mathrm{ERH}=\exp\left[\frac{\exp\left[p_{1}+p_{2}T\right]}{-{\left(100\mathrm{EMC}\right)}^{p_{3}}}\right]$
Modified Henderson	$\mathrm{ERH}=1-\exp\left[\frac{-p_{1}\left(T+p_{2}\right)}{{\mathrm{EMC}}^{-p_{3}}}\right]$
Modified Oswin	$\mathrm{ERH}={\left[1+\frac{{\left(p_{1}+p_{2}T\right)}^{p_{3}}}{{\mathrm{EMC}}^{p_{3}}}\right]}^{-1}$

EMC and ERH in decimal; *T* is temperature (°C); {$p_{1}$, $p_{2}$, $p_{3}$}: the parameters that need to be determined through fitting tasks.

### Thermodynamic desorption characteristics

The differential molar quantity of the isosteric heat of sorption can be obtained from the temperature dependence of the fitted sorption equations, according to Eqn (4), derived from the Clausius–Clapeyron equation:^8^, ^11^

(4) $$\frac{\partial \;\ln\left(\mathrm{ERH}\right)}{\partial \;\left(\frac{1}{T^{\mathrm{abs}}}\right)}=-\frac{Q_{\mathrm{st}}-L_{v}}{R}=-\frac{q_{\mathrm{st}}}{R}=-\frac{∆H}{R}$$

Notably, the critical term in this equation is $\frac{\partial \;\ln\left(\mathrm{ERH}\right)}{\partial \;\left(\frac{1}{T^{\mathrm{abs}}}\right)}$, i.e. the derivative of $\ln\left(\mathrm{ERH}\right)$ with respect to $\frac{1}{T^{\mathrm{abs}}}$. This term can be determined in two different ways. One way is the graphical differentiation, which ends up with a temperature‐independent expression for $q_{\mathrm{st}}$. The derivative of $\frac{\partial \;\ln\left(\mathrm{ERH}\right)}{\partial \;\left(\frac{1}{T^{\mathrm{abs}}}\right)}$ at a specific EMC will be found as the slope of the line redrawn in a diagram with $\frac{1}{T^{\mathrm{abs}}}$ on the *x*‐axis and $\ln\left(\mathrm{ERH}\right)$ on the *y*‐axis. Another way of determining $\frac{\partial \;\ln\left(\mathrm{ERH}\right)}{\partial \;\left(\frac{1}{T^{\mathrm{abs}}}\right)}$ is to employ a mathematical expression of a well‐fitted sorption equation. The sorption equation of ERH as a function of both EMC and temperature will be used to determine $q_{\mathrm{st}}$ analytically. The latter method was implemented to investigate the temperature dependency of $q_{\mathrm{st}}$.The Gibbs–Helmholtz equation was also considered to calculate the change in the molar differential entropy of desorption (∆S).^16^ Equation (6) relates the changes in both enthalpy and entropy during the water sorption process, as presented in Eqn (5):^30^

(5) $$\ln\left(\mathrm{ERH}\right)=-\frac{∆H}{R}\left(\frac{1}{T^{\mathrm{abs}}}\right)+\frac{∆S}{R}$$

(6) $$∆H=T_{\beta }∆S+∆G_{\beta }$$

Linear regression was conducted to determine the slope (−Δ*H*/*R*) and the intersection of the line (Δ*S*/*R*) at a specific moisture content of the material. This procedure was repeated at various EMC values, using each of the sorption equations, to show the dependence on the moisture content for both Δ*H* and Δ*S*. The compensation theory was considered as it proposes a linear relationship between ∆H and ∆S, as shown in Eqn (6).^31^, ^32^, ^33^ The isokinetic temperature $T_{\beta }$ and differential Gibbs free energy $∆G_{\beta }$ (J mol^−1^_water_) were calculated using linear regressions through the pairs of (∆H, ∆S) at certain moisture content levels.^11^

According to the procedure described in a previous report,^34^ given the existence of a linear relationship between pairs of (∆H, ∆S), the isokinetic temperature should be compared with the harmonic mean temperature (*T*_hm_) (Eqn (7)) to validate the theory of compensation:

(7) $$T_{\mathrm{hm}}=\frac{5}{\sum_{i=1}^{5}1/{T^{\mathrm{abs}}}_{i}}$$

Therefore, if $T_{\beta }$ ≠ *T*_hm_, there will be a linear compensation pattern between Δ*H* and Δ*S*. Furthermore, the desorption process is enthalpy‐controlled if $T_{\beta }$ > *T*_hm_. In the same way, $T_{\beta }$ < *T*_hm_ denotes the existence of an entropy‐controlled process. Linked to satisfying this theory, further analytical calculations were made to assess the net isosteric heat of desorption ($q_{\mathrm{st}}$) as a function of EMC and temperature. According to a previous report,^7^ the obtained isosteric heat of desorption ($Q_{\mathrm{st}}$) will be used to calculate the energy efficiency in flatbed dryers in a set amount of the input energy rate (${\dot{Q}}_{\text{in}}$) as shown in Eqn (8):

(8) $$\eta =\frac{{\dot{m}}_{w}\times Q_{\mathrm{st}}\times W_{w}}{{\dot{Q}}_{\text{in}}}$$

where $W_{w}$ is the weight of a water molecule, equal to 0.018 kg mol^−1^, and ${\dot{m}}_{w}$ is the water evaporation rate (kg h^−1^) defined as in Eqn (9). Knowing the initial weight ($W_{\mathrm{ini}}$) and the moisture content ($X_{\mathrm{ini}}$) of the batch, the water evaporation rate can be calculated as in Eqn (9) for each time interval ∆t, where ∆X denotes the obtained reduction in moisture content during that time interval:

(9) $${\dot{m}}_{w}=\frac{W_{\mathrm{ini}}}{1+X_{\mathrm{ini}}}\left(\frac{∆X}{∆t}\right)$$

## RESULTS AND DISCUSSION

### SEM analysis

**Figure 2:**
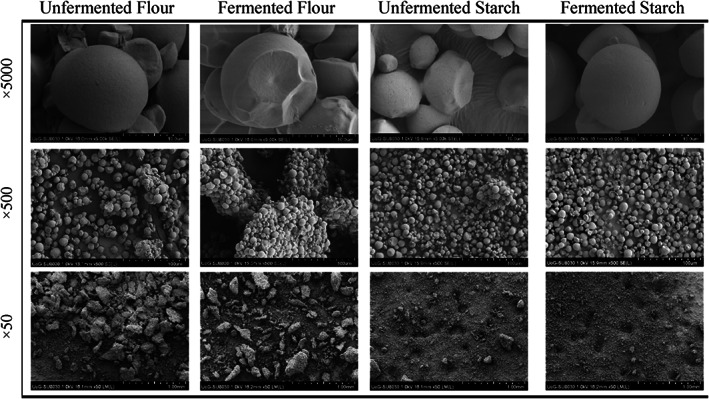
Scanning electron micrographs of unfermented flour, fermented flour, unfermented starch and fermented starch at three levels of magnification (50×, 500× and 5000×).

SEM photographs of the cassava granules are presented in Fig. 2. No differences were observed between the samples that underwent the desorption process at different air temperature levels. This observation was expected because all the samples had been thoroughly dried in the vacuum chamber in the coating stages. SEM images at 5000× scale suggest similar morphology for four products, captured as smooth, round and truncated spherical or ellipsoidal granules. This finding acknowledged the granular morphologies of cassava reported in other works.^18^, ^19^ However, some differences emerge at smaller magnification levels of 500× and 50× scales, where fermentation accentuates the cluster formation, particularly in flour samples. This caused the formation of coarser accumulation of starch granules. Nonetheless, the flour samples possessed more agglomerates than the starch samples in size and population.

### Desorption isotherms

#### Experimental results

**Figure 3:**
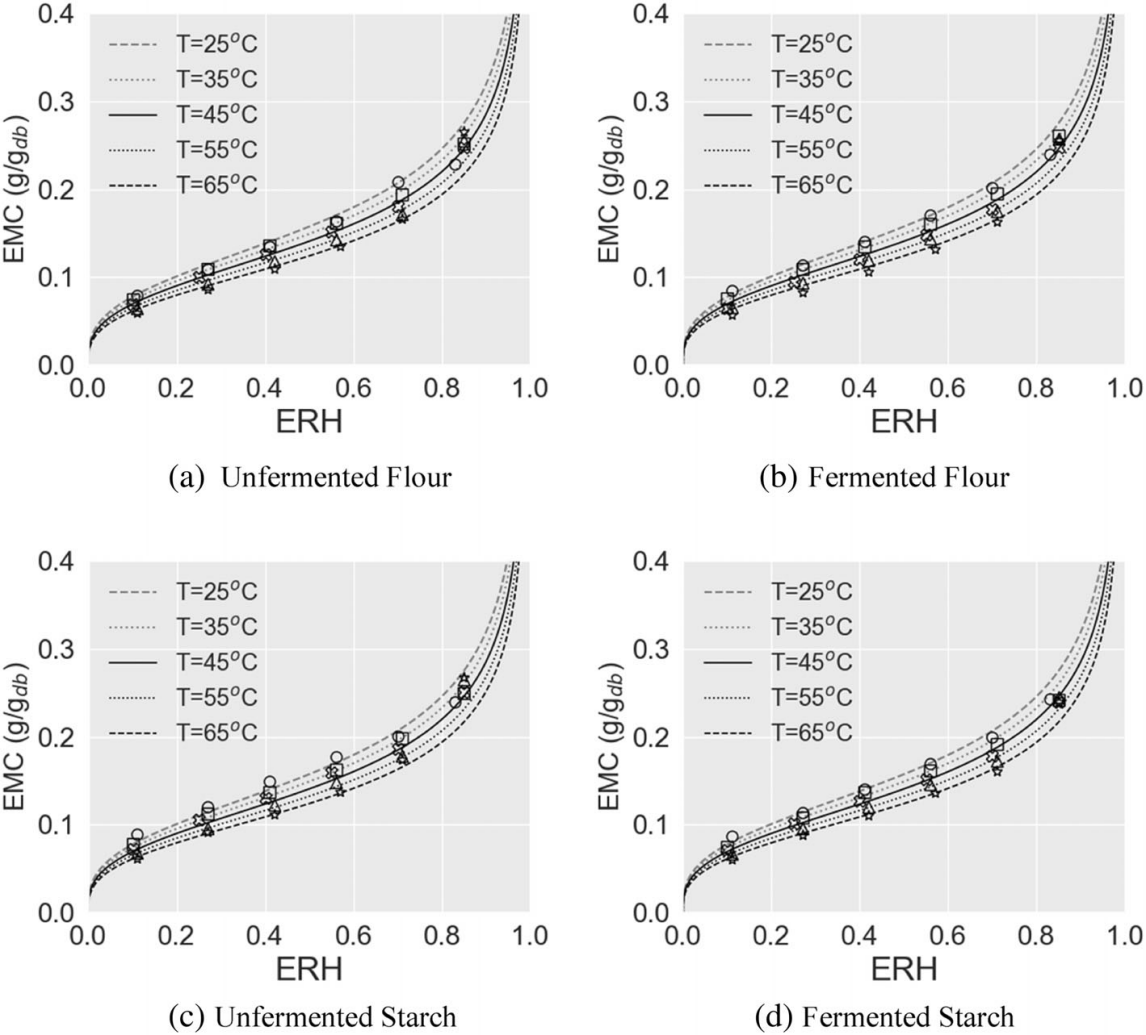
Experimental desorption data measured separately for (a) unfermented flour, (b) fermented flour, (c) unfermented starch and (d) fermented starch at five temperature levels, represented by ○ for 25 °C, □ for 35 °C, × for 45 °C, Δ for 55 °C and ☆ for 65 °C, along with mathematically predicted desorption isotherms as grey dashed line for 25 °C, grey dotted line for 35 °C, black solid line for 45 °C, black dotted line for 55 °C and black dashed line for 65 °C, obtained by the best‐fitted equation of the modified Oswin, as an example.

The measured desorption isotherm data are depicted in Fig. 3. EMC values were obtained from 0.057 to 0.268 kg kg^−1^ db using the automated gravimetric moisture sorption system at five levels of set relative humidity ranging between 0.10 and 0.85. According to the isotherm form classification,^8^ a typical sigmoidal form can best represent all the obtained desorption isotherms. This form is similar to the characteristic of the majority of biological products.^35^ The obtained desorption isotherms were comparable in values and patterns to those reported in other studies^23^, ^36^, ^37^ for similar cassava products. The results depicted in Fig. 3 suggest that the preparation methods, being neither flour nor starch nor using fermentation, did not cause perceptible differences in the desorption isotherms. This was acknowledged by analysis of variance (ANOVA) test results. Table 2 presents the ANOVA results, showing the dominant effects of ERH and temperature on the dependent variable of EMC. The independent variables’ combinational effect proved insignificant throughout the preliminary analysis. The main effect of ERH yielded an effect size of 0.963, indicating that ERH explained 96.3% of the variance in the desorption isotherms (*F*(5, 107) =1283.13, *P* < 0.001). For temperature levels, only 2% of the variance in the desorption isotherms was explained by this factor (*F*(4, 107) = 32.847, *P* < 0.001). However, less than 1% of the variance in the desorption isotherms is associated with the product types; the effect of this factor is still significant on the isotherms at a significance level of 0.05 (*F*(3, 107) =3.1404, *P* = 0.02836). These conclusions agreed with the earlier visionary deductions made in Fig. 3. The current findings and results published previously^36^ may suggest that neither the preparation procedure nor the variety significantly affects the sorption isotherms of pulverised cassava grits.

**Table 2:** ANOVA test results examining the measured EMC values as a function of product type, ERH and temperature

Factor	df	SS	MS	*F‐*value	*P*‐value	*η* ^2^
ERH level	5	0.40384	0.080767	1283.1268	<0.001	0.963
Temperature level	4	0.00827	0.002068	32.8468	<0.001	0.020
Product type	3	0.00059	0.000198	3.1404	0.0284	0.001
Residual	107	0.00674	0.000063			

#### Desorption isotherm models

Table 3 shows the fitting characteristics indicating how the sorption equations reproduce ERH data well. The coefficient of determination (*R* ^2^) can be interpreted as the proportion of the variance in the predicted ERH values attributable to the variance in the actual ERH values. In agricultural engineering, a good fit is assumed when MRPE is below 10%.^38^ SEE made another judgement to choose the best possible quality of fit. The main difference between MRPE and SEE is that SEE puts a heavier weight on larger errors. Thus SEE is more sensitive to outliers compared to MRPE. The modified Oswin and the modified Chung–Pfost gave noticeably better fits than the others at all temperatures throughout the entire range of studied ERH. This finding can be seen in Fig. 3, where only constructed desorption isotherms with the modified Oswin model are illustrated, for brevity.

**Table 3:** Accuracy of fit and estimated parameters of the empirical ASAE sorption equations (Table 1) in reproducing the measured desorption isotherms

Sorption equation	Model fit index		Model parameter		
	SEE	MRPE	*p*_1_	*p*_2_	*p*_3_
Modified Chung–Pfost	0.0259	5.96	533.4351 ± 26.0750	15.6235 ± 0.2109	38.9053 ± 3.9891
Modified Halsey	0.0344	10.71	5.5667 ± 0.1142998	−0.0127 ± 0.0008163	2.0859 ± 0.0380959
Modified Henderson	0.0371	9.52	0.53096 ± 0.06678	41.87321 ± 6.28502	2.15928 ± 0.04235
Modified Oswin	0.0236	5.12	0.1783 ± 0.00172	−0.0008338 ± 0.00003531	3.109 ± 0.04018

Moreover, the parameters were predicted as ranges, represented by mean and standard deviation values. As an average for all three parameters, the smallest coefficient of variation (the ratio of standard deviation to mean) was found for the parameters of the modified Oswin model. The predicted parameters for the modified Henderson equation had the highest uncertainty compared to the other models. If MRPE is less than 10%, the corresponding model could be considered a good fit.^39^ This criterion clarifies that the modified Halsey models violated this threshold marginally. Even though the modified Henderson showed less than 10% of MRPE, it does not show a normal distribution of residuals. Therefore, it provided evidence that the predictions obtained by the modified Henderson model may not be as reliable as the other models.

Meanwhile, the modified Oswin and the modified Chung–Pfost models showed promising MRPE values of 5.12 and 5.96, respectively. Nonetheless, modified Chung–Pfost also appeared reliable in having normal residual distribution. They also showed minor SEE errors equal to 0.024 and 0.026, respectively. These values were followed by 0.034 and 0.037, respectively, for modified Halsey and modified Henderson models. Overall, the modified Oswin model was highlighted to yield the most optimal fit, followed by the modified Chung–Pfost and modified Halsey models.

### Thermodynamic characteristics

**Figure 4:**
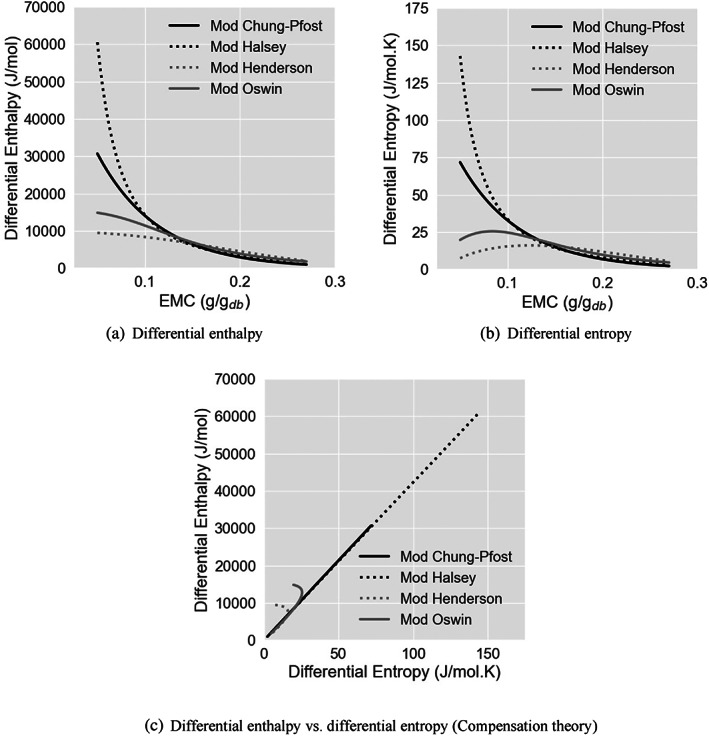
Differential enthalpy (a) and entropy (b) during desorption process, obtained by using different ASAE empirical equations (modified Chung–Pfost: black solid line, modified Halsey; black dotted line, modified Henderson; grey dash‐dot line; modified Oswin: grey solid line) along with their relationship as the justification of the compensation theory (c).

The thermodynamic parameters provide insights into the theoretical interpretations for food matrix–water interactions.^11^, ^17^ Figure 4(a,b) depicts the variation of ∆H and ∆S with respect to the EMC values by rewriting Eqn (4), where the crucial role of the applied desorption equation was highlighted. As the desorption progressed and EMC decreased, the water molecules were tightly bound on the surfaces of the cassava particles. This situation demands higher energy to break the bonds and release the water and thus will be marked by higher levels of ∆H. At the same time, differential entropy quantifies the number of available sorption sites corresponding to a specific energy level.^40^

According to the mathematical modelling results, more variations in the low moisture content range are shown in Fig. 4. Reviewing the residual distribution in predicting actual ERH values at various moisture content levels showed that the range of variation in ∆*H* and ∆*S* is not noticeably influenced by the fitting quality of the used sorption equation. In Fig. 4, two different behaviours were found in the graphs. This dissimilarity split the applied desorption equations into two groups. The first group was composed of the modified Chung–Pfost and the modified Halsey models, depicted as black graphs in Fig. 4, while modified Henderson and modified Oswin models formed the second group, depicted as grey graphs in Fig. 4.

The modified Oswin model and the modified Chung–Pfost model, which have already shown the best fitting adequacy features, were placed in different groups. This therefore revealed a severe concern about whether the fitting adequacy is sufficient to proceed with further thermodynamic characterisations. Accordingly, the compensation theory was employed, which appeared to be valid for most of the sorption studies on food materials, particularly starchy food materials.^8^, ^9^, ^30^, ^31^, ^41^, ^42^, ^43^ The premise of compensation theory affirms that the more substantial the intermolecular interaction, the more significant the reduction in the configurational freedom, and hence the more significant the order of the system.

Figure 4(c) shows the parallel changes in ∆H and ∆S, where using the first group of desorption equations led to a linear relationship of ∆H– ∆S throughout the desorption process. On the other side, for the second group, this linear relationship occurred only in the first half of the desorption process, followed by an upward twisting towards the end of the process. This behaviour could be traced in Fig. 4(a,b), where the increasing rate of both ∆H and ∆S started to change at some specific amounts of EMC as the desorption process progressed towards the monolayer sorption area. The compensation theory helps confirm whether the increase in enthalpy, representing a generation of larger order over disorganisation, co‐occurs with a proportionate increase in the differential entropy, representing greater freedom of the molecules in the food. On the other hand, the second group showed a tendency of flattening for ∆H, while Δ*S* graphs behaved slightly differently, as they showed a decreasing pattern instead of flattening at some points in the second half of the desorption process. In comparison, the first group resulted in an exponentially increasing trend for both ∆H and ∆S with respect to decreasing EMC. Several references can be found in the literature,^21^, ^42^, ^44^ supporting the exponential increase of Δ*H* and Δ*S* with decreasing EMC during the desorption process.

It is critical to corroborate that the emerged linear relationships (in Fig. 4c) reflect merely the thermodynamical properties of the product, not the propagation of the experimental error. For this reason, the authenticity of the compensation theory was investigated according to the procedure described previously.^34^ According to Eqn (6), for the current research's desorption processes, a value of 44.13 °C was found for the harmonic mean temperature. The lower thresholds of $T_{\beta }$ at 95% confidence level, 154.6 °C for the modified Chung‐Pfost equation and 150.2 °C for the modified Halsey equation, were significantly higher than the harmonic mean temperature of 44.13 °C. Therefore, it was concluded that the compensation theory was relevant, and the desorption process was controlled only by enthalpy. $T_{\beta }$ is a helpful quantity suggesting the temperature at which the kinetic constant should be the same regardless of the environmental variable.^33^ McMinn *et al*. obtained $T_{\beta }$ values between 93.65 and 193.65 °C for starch‐containing materials^9^ with the obtained values here. The enthalpy‐driven nature of the desorption process indicates that the amount of energy required for removing water attached to the constituents of the cassava granules is greater than the energy consumed for reorganising the system molecules. Similar results were previously obtained for sweet potato,^43^ starch,^9^ oat flour and biscuits,^31^ and potato and sweet potato flakes.^42^ The near‐zero values obtained for $∆G_{\beta }$ denote that the system would be in equilibrium at the isokinetic temperature. Regarding the high content of starch in cassava, a similar behaviour can be expected considering previous studies for starchy products.^30^, ^45^

**Figure 5:**
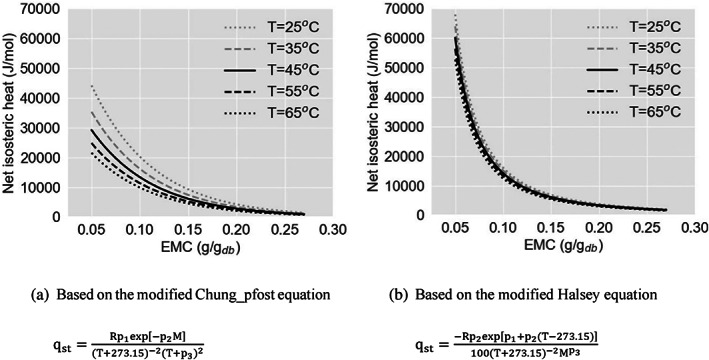
Temperature dependency of the net isosteric heat of desorption during the desorption process (grey dotted line: 25 °C; grey dashed line: 35 °C; black solid line: 45 °C; black dashed line: 55 °C; black dotted line: 65 °C) obtained by using empirical ASAE equations of (a) modified Chung–Pfost and (b) modified Halsey.

The mathematical equations of the modified Chung–Pfost and the modified Halsey were selected for predicting the heat of sorption as a function of the product moisture content and drying air temperature by substituting into Eqn (3). Isosteric heat of desorption ($Q_{\mathrm{st}}$) is a vital quantity to estimate the energy requirements of the drying processes and provides essential information on the state of water in foodstuffs.^17^, ^24^, ^40^ As Eqn (3) suggests, $Q_{\mathrm{st}}$ can be obtained by adding the heat of vaporisation of pure water at saturation pressure, placed between 44.200 kJ mol^−1^ at 20 °C and 42.030 kJ mol^−1^ at 70 °C. The net isosteric heat of desorption ($q_{\mathrm{st}}$) depends on the state of water held by the solid material, which is a function of temperature by itself. Following the Clausius–Clapeyron equation, $q_{\mathrm{st}}$ was calculated analytically as a function of both EMC and temperature. The derived relationships are written and illustrated in Fig. 5.

Results proved that higher energy would be needed at lower temperatures. The temperature effect was more sensible in the second half of the desorption process compared to the first half. However, the graphs obtained by the modified Halsey model showed a lower sensitivity to the temperature (Fig. 5b) than the modified Chung–Pfost model (Fig. 5a). For instance, at 0.1 kg kg^−1^ db, $q_{\mathrm{st}}$ varied between 9.17 and 19.97 kJ mol^−1^ according to the modified Chung–Pfost model, whereas the modified Halsey model led to a range of 12.19–15.73 kJ mol^−1^ in the temperature range of 25–65 °C. Once again, without applying the compensation theory as a justification criterion, one may use the best‐fitted desorption equation of modified Oswin (see fitting indices in Table 3), which would lead to the peculiar behaviour of having higher net isosteric heat of desorption at higher temperature in the last half of desorption process.

The results showed that all EMC led to positive $q_{\mathrm{st}}$ values, indicating that not only the process of water desorption was endothermic, but it was also necessary to have a heating source supplying energy more than what is needed for the vaporisation of pure water to continue the desorption process. At lower EMC, exponentially increasing values were estimated for $q_{\mathrm{st}}$, indicating that the adsorbent–adsorbate interactions must be more significant than the interactions among water molecules. Fasina *et al*. observed a very similar trend for roasted cassava products. Comparable results were also found in the literature for similar starchy products,^12^, ^40^, ^42^ and some cassava products.^17^, ^21^, ^23^, ^24^, ^44^ For example, for roasted milled powder of cassava,$q_{\mathrm{st}}$ was found to vary from 39.47 kJ mol^−1^ at 0.01 kg kg^−1^ db to 4.92 kJ mol^−1^ at 0.2 kg kg^−1^ db^23^ – very close to the results obtained by either Koua et al.^24^ or using the modified Halsey model here (Fig. 5b). Similar values for the unroasted product were found to vary from 26.98 kJ mol^−1^ at 0.01 kg kg^−1^ db to 4.97 kJ mol^−1^ at 0.2 kg kg^−1^ db,^23^ which was close to the results obtained by the modified Chung–Pfost model (Fig. 5a).

Overall, as seen in Table 4, three consecutive criteria were considered to compare the capability of the empirical sorption equations suggested by the ASAE standard in studying the desorption behaviour of cassava products. It was found that the modified Chung–Pfost equation showed the best features to represent the desorption behaviour of understudied cassava products. The results proved that one must not only rely on the fitting adequacy of sorption equations, as the modified Oswin model with the best‐shown ability to fit over desorption isotherms (see Table 3) failed to fulfil the compensation theory criteria.

**Table 4:** Comparing the capability of different empirical ASAE sorption equations in showing the desorption behaviour of cassava products

Criterion		Modified Oswin	Modified Chung–Pfost	Modified Halsey	Modified Henderson
Fitting desorption isotherms	Table 3	●●●●	●●●	●●	●
Fulfilling compensation theory	Fig. 4(c)	—	●●●●	●●●●	—
Reflecting temperature dependency of the isosteric heat of desorption	Fig. 5	—	●●●●	●	—

### Technological relevance of the findings

Energy efficiency is an essential characteristic of dryers, which is of great interest for designers and decision‐makers as it directly relates to drying costs. Typical energy efficiency values are 41.3% and 58.7% for a fixed‐bed dryer and a pneumatic dryer, respectively. These were values obtained for drying wet cassava grits (initial moisture content of 0.87 kg kg^−1^ db) in West and sub‐Saharan Africa regions.^7^

**Figure 6:**
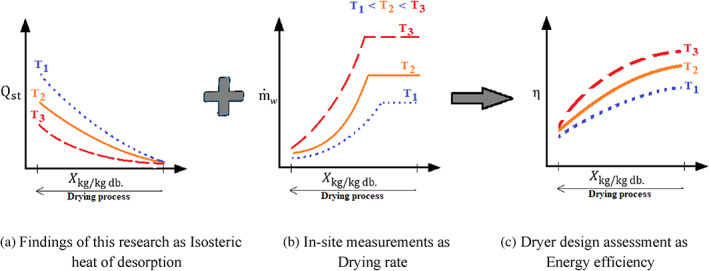
Schematic diagrams demonstrating how the results can be used in assessing the energy efficiency of cassava dryers, showing the temperature effect (lower temperature in dotted blue lines, middle temperature in solid orange lines and higher temperature in dashed red lines). (a) Findings of the current study (depicted in Fig. 5); (b) drying rate curves that can be obtained according to Eqn (8) by a set of in‐site measurements of moisture content throughout the drying process; (c) drying efficiency curves calculated with Eqn (7).

According to Eqn (7), ${\dot{m}}_{w}$ is key in determining the energy efficiency expressed by η. Data collected in the literature on producing industrial cassava flour from grated unfermented cassava mash showed an explicit dependency on the initial moisture content, drying air temperature, airflow rate and the combination of these factors.^46^ The current study's findings can contribute to evaluating the energy efficiency of a dryer as the drying process progresses. Schematic graphs in Fig. 6 show how the obtained results can be used in companion with a set of in‐site moisture content measurements to determine the variation of energy efficiency of the cassava dryers throughout the drying process. The variation of moisture content (∆X) at each specific time interval (∆t) will determine the water evaporation rate (${\dot{m}}_{w}$) at that part of drying (Eqn (8)).

These findings will provide enough information for a designer to make wise decisions about the working conditions of a dryer at different stages of the drying process. Figure 5 also shows how adjusting the drying air temperature might change the energy efficiency, as it impacts both input thermal energy and drying rate. Knowing the temperature dependency of $q_{\mathrm{st}}$ is vital for finding the most optimised drying air temperature. Moreover, the relevant data in Odetunmibi et al.^46^ shows a significant decrease in the drying rate by reducing the air temperature. Likewise, lower enthalpy is expected for the air with lower temperatures, resulting in lower thermal energy input. Hence choosing the optimal combination of the airflow rate and temperature can secure an acceptable range of drying efficiency throughout the drying process.

## CONCLUSIONS

The application of four well‐known empirical sorption equations suggested by the ASAE standard for predicting the isosteric heat of sorption for cassava products was investigated based on experimental data. An automated gravimetric moisture sorption system was implemented to measure desorption data for cassava flour and starch, either fermented or unfermented. The obtained desorption isotherms showed no statistical differences between different cassava products. This similarity also agrees with the SEM exploration of the samples.

The ASAE empirical sorption equations were first examined based on their fitting adequacy over the measured isotherms. Then, their suitability towards fulfilling the compensation theory was also studied during further thermodynamic analysis. Finally, the ability of the models to reflect the temperature dependency of net isosteric heat of sorption was investigated.

The results of this research highlight the suitability of the modified Chung–Pfost equation for investigating the desorption behaviour of cassava products, particularly for dryer design purposes. It was shown how the findings could contribute to the evaluation of the cassava dryers. It is crucial from the techno‐economic perspective to maximise the energy efficiency throughout the drying process for the ultimate benefit of designers and stakeholders.

## AUTHOR CONTRIBUTIONS STATEMENT

**Hamed J Sarnavi**: conceptualisation, methodology, visualisation, writing original draft. **Marcelo Precoppe**: conceptualisation, methodology, investigation, resources, writing – review and editing, supervision, project administration. **Pablo García‐Triñanes**: conceptualisation, methodology, investigation, resources, writing – review and editing, supervision, formal analysis. **Arnaud Chapuis**: literature review, writing – review and editing. **Thierry Tran**: conceptualisation, validation, writing – review and editing, funding acquisition. **Michael SA Bradley**: methodology, writing – review and editing, supervision. **Joachim Müller**: conventionalization, resources, funding acquisition, supervision.
